# The Effect of Doxycycline on Achilles Tendon Repair in a Rat Model

**DOI:** 10.5704/MOJ.2011.024

**Published:** 2020-11

**Authors:** A Sobhani-Eraghi, M Panahi, A Shirani, H Pazoki-Toroudi

**Affiliations:** 1 Department of Orthopaedic Surgery, Iran University of Medical Sciences, Tehran, Iran; 2 Department of Pathology, Iran University of Medical Sciences, Tehran, Iran; 3 Department of Medicine, Iran University of Medical Sciences, Tehran; 4 Physiology Research Center, Iran University of Medical Sciences, Tehran, Iran; 5 Department of Physiology, Iran University of Medical Sciences, Tehran, Iran

**Keywords:** doxycycline, achilles tendon, tendon repair, matrix metalloproteinase

## Abstract

**Introduction:**

Doxycycline is a commonly used antibiotic that is also a potent inhibitor of matrix metalloproteinase (MMPs). The use of doxycycline in repairing tendon lesions has been previously investigated and conflicting findings have been reported on its effectiveness. In this study, we sought to evaluate the effects of exposure to doxycycline on Achilles tendon repair.

**Materials and Methods:**

Twenty healthy rats of the same breed and gender were randomly assigned to two groups of sham, and Doxycycline group therapy. The rats underwent a surgical intervention in which a 2mm incision was performed on the lateral sides of the right Achilles tendons. The treatment group received oral gavage administrations of 50mg/kg/day of doxycycline for 30 days. After this duration, tissue samples were taken from the site of the injuries, which were then histologically evaluated for alignment of the collagen fibres, inflammation reaction, cellular density, and fibroblastic activity.

**Results:**

The histological assessment of the tissue samples, revealed significant changes in the repaired tissues of the treatment group in comparison to the sham group; namely more irregularity in the alignment of the collagen fibres, increased cellular density, and increased fibroblastic activity. However, only the alignment of the collagen fibres reached the statistical significance.

**Conclusion:**

The results of this study indicate that exposure to doxycycline may result in the improvement of repair of the Achilles tendon injuries, especially collagen filament integrity.

## Introduction

Achilles tendon injuries are devastating and could have potentially unfavorable outcomes with regard to return to normal function^1,2^. Various studies have shown that inoperative treatment may lead to similar outcomes to reconstructive surgery, with early functional rehabilitation important for both treatment options. Despite these benefits of operative treatment of Achilles tendon ruptures, surgical complications exist^3^. Tendon repairs are different based on the tendon junction, anatomic site, load type, and relationship with the affected joint. Over the past 40 years, the prevalence of Achilles tendon rupture has steadily increased. The incidence of Achilles tendon rupture in the general population is approximately 5 to 10 per 100,000 but may be higher in some areas and populations. More than 80% of tears occur in recreational sports^4,5^.

Collagenase or matrix metalloproteinases MMPs have been found to play an essential role in the degradation of related enzymatic matrix with tendon damage and disease. MMPs are natural zinc-dependent proteases that have the potential to break down almost all components of the extracellular matrix. After tendon rupture, increased MMPs activity and increased matrix efficiency will cause the collagen network to disappear^6,7^.

Doxycycline is a member of the tetracyclines family that is commonly used as a broad-spectrum antibiotic. In addition to the antimicrobial effects, the tetracycline family can also inhibit MMPs. Of the approved tetracyclines, doxycycline has been shown to be the most potent MMPs inhibitor. Moreover, in vitro studies have also shown that doxycycline has the ability to inhibit collagen synthesis^8,9^. The use of doxycycline in repairing tendon lesions has been previously investigated and conflicting findings have been reported on its effectiveness. In a study on rotator cuff injury, doxycycline has shown a positive effect on tendon repair and histological findings^10^. Two other studies, on the other hand, reported contradicting results ofdoxycycline effect on the process of repairing Achilles tendon lesions through mechanical evaluation and stretch tolerance^11,12^.

Considering the above mentioned and the role of doxycycline in inhibiting MMPs and reducing collagen synthesis, this study aimed to assess the effects of daily oral administration of doxycycline on Achilles tendon healing.

## Materials and Methods

The experiment was performed on 20 adult male Wistar rats weighed 200–250g and aged 120-140 days; the rats were maintained under a standard condition with ambient temperature (22±2°C) and reverse light–dark cycle (12/12h) with food and water available ad libitum. Experiments were conducted in accordance with the guidelines of the national ethics committee and the institutional animal ethics committee of Iran University of Medical Sciences. The rats were provided by the Pasteur animal centre, Tehran.

Rats were randomly divided into two groups of 10 in the control and treatment groups. After exposure, transverse incision was given to the Achilles tendon 2mm from the external side for all animals in both groups Then in the treatment group, doxycycline was administrated one day after surgery with a daily dose of 50mg/Kg for 30 days through oral gavage.

Finally, at the evaluation day, animals were killed by CO_2_ asphyxiation and tissue samples were examined for collagen status, inflammatory response, cell density and fibroblast activity by hematoxylin and eosin (H&E) staining.

All surgeries were performed by the leading author on the right lower extremity. The animals were anaesthetised with ketamine (100mg/kg) and xylazine (10mg/kg) by intra peritoneal injection. As for the procedure, Achilles tendon was exposed through a 1.5cm skin and deep fascia-splitting incision in the posterior surface of the leg, and then in 5mm proximal to Achilles tendon attachment to the calcaneus, 2mm transverse incision was made in Achilles tendon. Thus, tendon defect was created and left unsecured to regenerate. Rats were under observation for two hours after surgery, following which they returned to their living place and allowed to start normal activities.

Specimens were prepared from 1cm long Achilles tendon proximal to the attachment to the calcaneus. Tendons were fixed in formalin solution and stained with hematoxylin and eosin (H&E). Specimens were assessed for inflammation reaction, collagen fibres condition, cellular density, and fibroblast activity under a light microscope.

Inflammatory reaction was evaluated based on degree of inflammatory cell infiltrations (low, medium, high). Collagen fiber condition was evaluated from the points of continuity, consistency, order, thickness, density, and orientation (parallel or nodular).

Cellular density and fibroblast activity was evaluated from the point of fibroblast numbers and its shapes (low, medium, high). To avoid bias, all histologic evaluation was performed by a pathologist who was blind to the group of cases.

All data analysis was conducted using SPSS version 18. Descriptive data were reported as a mean± standard deviation for Continuous values and frequency for categorical values. Chi-Square was employed to interpret data. P values less than 0.05 were considered statistically significant.

## Results

**Fig. 1:**
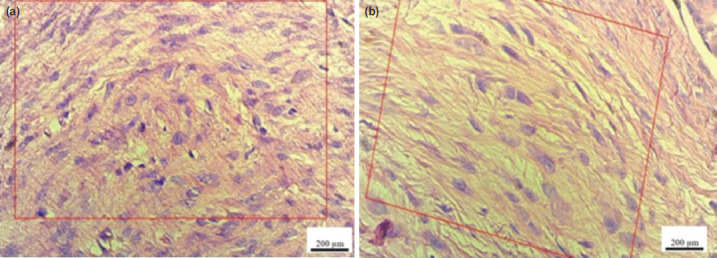
(a) Tissue samples of collagen fibres in control group and (b) doxycycline group. In the control group, most of the collagen strands were nodular oriented, whereas in the doxycycline group the collagen filaments were almost parallel. (H&E magnification 40×, scale bar 200 μm).

In this study, tissue samples prepared from Achilles tendon in rats were divided into two groups of control and doxycycline group based on four criteria (collagen status, inflammatory response, cell density and fibroblast activity). Histological evaluation of the collagen showed that in the control group, the collagen fibres were partially disrupted and the order and consistency disappeared. The collagen filaments density and thickness also decreased and their orientation was nodular, but in the doxycycline recipient group the orientation of the collagen fibres was almost parallel and continuous, and thickness of the strands increased compared to the control group (Fig. 1). Table I also shows that the number of animals with almost parallel strands in the treatment group was higher than the control group (P = 0.022).

**Table I:** Collagen deposition condition

Group		N (%)	p-value
Sham	Parallel	2 (20)	0.022
	Almost parallel	2 (20)	
	Nodular	6 (60)	
Doxycycline	Almost parallel	10 (100)	

**Fig. 2:**
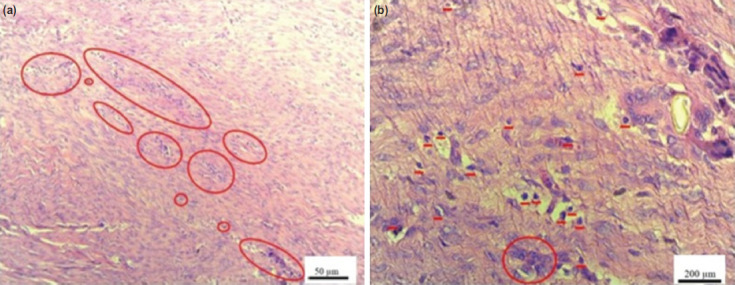
(a) Tissue samples of inflammatory cell infiltrates in control and (b) doxycycline treatment groups. In the control group the highest inflammatory cell infiltration and in the treatment group the inflammatory cell infiltration decreased compared to the control group. (H&E magnification A 10 ×, scale bar 50 μm, B 40×, scale bar 200 μm).

Histological evaluation showed that the highest level of inflammatory cell infiltration was observed in the control group (Fig. 2). However, the difference in the number of rats based on the inflammatory reaction was not statistically significant(P = 0.891) (Table II).

**Table II:** Inflammation reaction, cellular density and fibroblast activity in Sham and Doxycycline groups.

Group		Low N(%)	Medium N(%)	High N(%)	p-value
Inflammation reaction	Sham	2 (20)	2 (20)	6 (60)	0.891
	Doxycycline	6 (60)	4 (40)	0	
Cellular density	Sham	0	4 (40)	6 (60)	0.891
	Doxycycline	6 (60)	4 (40)	0	
Fibroblast activity	Sham	0	4 (40)	6 (60)	0.891
	Doxycycline	6 (60)	4 (40)	0	

**Fig. 3:**
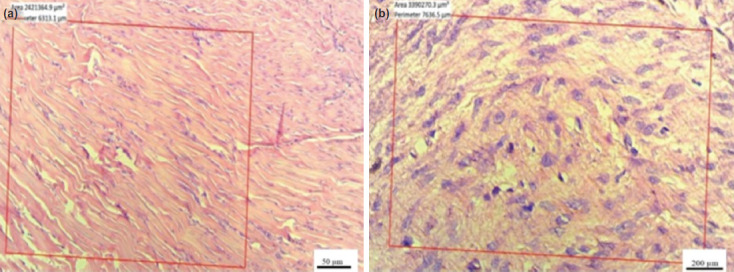
(a) Tissue samples of proliferation and cell density in the control and (b) doxycycline treatment groups. In the control group, cells with dense, oval-shaped nuclei are seen, indicating a low activity of fibroblasts, which increased the rate of fibroblasts in the treatment group compared to the control group. (H&E magnification A 10×, scale bar 50 μm and B 40×, scale bar 200 μm).

As shown in (Fig. 3), the examination of cell density and number of fibroblast cells showed that tissue samples in the control group demonstrated cells with dense, oval-shaped cells, indicating low activity of fibroblasts. On the contrary, cells with round, large, and heterogeneous nuclei were seen in the treatment group, indicating high levels of fibroblast activity. This observation, however, did not reach a statistical significance (P=0.891)(Table II).

## Discussion

Achilles tendon rupture is one of the most common injuries that have unpredictable effects on the return to normal function. The results of studies have shown that non-surgical therapies can have the same results as surgery-based therapies^13^. Numerous studies have shown the important role of collagenases or matrix metalloproteinase or MMPs in enzyme matrix fixation cascades associated with tendon injury and repair^7,14^.

In our study, the effect of doxycycline as the most potent inhibitor of MMPs was examined and it was shown that in animals receiving doxycycline, collagen filaments had increased order, attachment, and thickness to the affected group.

Based on the histological evaluation of the samples, although less severe inflammatory reaction, more cell density, and fibroblastic activity were evident in the treatment group, none of the aforementioned were significantly different from that of the sham group. Our findings on the 30-day period of doxycycline administration showed neither significant improvement nor impairment in Achilles tendon injury.

In general, tendon repair requires at least three separate but related processes: fibroblast cell proliferation, collagen fibril production and synthesis, and fibrils alignment with the longitudinal axis of the tendon. In the healing process, replacement of the tendon with fibrotic tissue is not good because the fibrotic tissue has gradually expanded and cannot withstand the stress of the movements and pressures on the leg muscles; and in the absence of complete and correct recovery in performance, the results are unpredictable^1,2^.

Tendon degeneration is an active cellular process that may be due to the lack of control of MMP activity in response to repeated injury or mechanical stress. After tendon injury and rupture, increased MMP activity is associated with impaired collagen network quality^15,16,17^.

Studies have shown that MMPs, upon activation, disrupt all connective tissue components, alter extracellular matrix, and cause a painful tendonopathy and tendon rupture. To inhibit MMP activity, cells secrete tissue matrix metalloproteinase (TIMP) inhibitors, which balance the MMPs and TIMPs, and exert control of the tendon component composition. Decreased MMP activity at basal level may decrease the pathological alteration of tissue and may lead to better tissue repair after injury^14,18^. The use of doxycycline in the repair of tendon lesions has been previously studied in vitro and in vivo, and contradictory findings have been reported on its efficacy. In a study of doxycycline use on rotator cuff lesion repair and it has shown positive effects in histopathologic evaluation^9,10^.

In two other studies, the effect of doxycycline on the process of healing of Achilles tendon injury has been investigated through mechanical evaluation and stretch tolerance, which have had inconsistent results^11,12^.

In a study by Kessler *et al* in 2014, they evaluated the effect of Doxycycline on Achilles tendon repair in both short-term (one week) and long-term (four weeks) administration. Authors reported that in the long-term administration group, the orientation of collagen strands was more organised than that of control group and the density of collagen fibrils increased, but in the short-term administration group, the orientation of the collagen strands was variable and irregular^19^. In another study by Pasternak *et al* Systemic administration of doxy for 14 days impaired healing of unrepaired Achilles tendons^11^. Additionally, the use of doxycycline following a tendon injury in rats led to improved tendon repair quality compared to control groups. Also, Doxycycline had a significant effect on reducing the dissociation of collagen strands but had no effect on the angles between strings^13^.

Concerning the role of MMPs in Bedi *et al*, in the evaluation of rotator cuff injury repair, increased metachromasis and improved collagen regulation were observed after doxycycline administration and decreased MMP-13 activity compared to the control group^10^. A study by Dong *et al*, on 10 rats for 12 weeks showed that oral doxycycline 10mg / kg / d significantly increased fibrosis cap thickness and reduced MMP expression, local and systemic inflammation, and decreased plaque vulnerability^8^. On the other hand, in the study of Nguyen *et al*, which investigated the effect of doxycycline on the Achilles tendon damaged with and without suture repair, doxycycline affected the quality of tendon repair, improved organisation of the damaged tissue, corrected orientation of collagen fibrils and reduced scattering and fiber disruption, as well as decreased MMP-3 expression and improved biomechanical properties of the sutured tendon compared to the untreated suture group^13^.

In this study, we used a dose of 50mg / kg of doxycycline, while in other studies a dose of 100-130mg / kg was used. In addition, changes in post-injury and post-rehabilitation strategies may affect the healing process of the Achilles tendon. In this study, animals were allowed to return to normal activity immediately after surgery. It seems that increasing the number of samples in the present study can eliminate the effect of chance and accident on the outcome of the study. One of the limitations of this study is the duration of drug use. So that increasing medication use for more than 30 days may have more healing effects.

## Conclusion

Our results indicate that daily oral administration of doxycycline improve tendon repair especially collagen filament integrity in a rat model although this finding should be replicated by larger studies.
